# Human Beta Papillomavirus Type 8 E1 and E2 Proteins Suppress the Activation of the RIG-I-Like Receptor MDA5

**DOI:** 10.3390/v14071361

**Published:** 2022-06-22

**Authors:** Stephanie Rattay, Martin Hufbauer, Christian Hagen, Bastian Putschli, Christoph Coch, Baki Akgül, Gunther Hartmann

**Affiliations:** 1 Institute of Clinical Chemistry and Clinical Pharmacology, University Hospital, University of Bonn, Gebäude 12, Venusberg-Campus 1, 53127 Bonn, Germany; christian.hagen@ukbonn.de (C.H.); bputschli@gmail.com (B.P.); ccoch@gmx.net (C.C.); gunther.hartmann@uni-bonn.de (G.H.); 2 Institute of Virology, Medical Faculty and University Hospital Cologne, University of Cologne, Fürst-Pückler-Str. 56., 50935 Cologne, Germany; martin.hufbauer@uk-koeln.de (M.H.); baki.akguel@uk-koeln.de (B.A.)

**Keywords:** human papillomavirus type 8 (HPV8), innate immunity, immune system, nucleic acid detection, virus host interactions, pattern recognition receptors, receptor signaling

## Abstract

Persistent infections of the skin with the human papillomavirus of genus beta (β-HPV) in immunocompetent individuals are asymptomatic, but in immunosuppressed patients, β-HPV infections exhibit much higher viral loads on the skin and are associated with an increased risk of skin cancer. Unlike with HPV16, a high-risk α-HPV, the impact of β-HPV early genes on the innate immune sensing of viral nucleic acids has not been studied. Here, we used primary skin keratinocytes and U2OS cells expressing HPV8 or distinct HPV8 early genes and well-defined ligands of the nucleic-acid-sensing receptors *RIG-I*, *MDA5*, *TLR3*, and *STING* to analyze a potential functional interaction. We found that primary skin keratinocytes and U2OS cells expressed *RIG-I*, *MDA5*, *TLR3*, and *STING*, but not *TLR7*, *TLR8*, or *TLR9*. While HPV16-*E6* downregulated the expression of *RIG-I*, *MDA5*, *TLR3*, and *STING* and, in conjunction with HPV16-*E7*, effectively suppressed type I IFN in response to MDA5 activation, the presence of HPV8 early genes showed little effect on the expression of these immune receptors, except for HPV8-*E2*, which was associated with an elevated expression of TLR3. Nevertheless, whole HPV8 genome expression, as well as the selective expression of HPV8-*E1* or HPV8-*E2*, was found to suppress MDA5-induced type I IFN and the proinflammatory cytokine IL-6. Furthermore, RNA isolated from HPV8-*E2* expressing primary human keratinocytes, but not control cells, stimulated a type I IFN response in peripheral blood mononuclear cells, indicating that the expression of HPV8-*E2* in keratinocytes leads to the formation of stimulatory RNA ligands that require the active suppression of immune recognition. These results identify HPV8-*E1* and HPV8-*E2* as viral proteins that are responsible for the immune escape of β-HPV from the innate recognition of viral nucleic acids, a mechanism that may be necessary for establishing persistent β-HPV infections.

## 1. Introduction

Human papillomaviruses (HPV) are small non-enveloped epitheliotropic DNA viruses with a circular genome of about 8000 bp. Currently, 441 HPV types are known to have co-evolved and persist in the human population (http://pave.niaid.nih.gov/ (accessed on 9 March 2022)) [1,2]. HPVs are phylogenetically grouped into five genera according to the DNA sequence homology of the *L1* gene (encoding the L1 capsid protein), namely alpha (α), beta (β), gamma, mu, and nu [3]. α-HPV types infect both cutaneous and mucosal epithelia and are further classified as either low-risk (LR-) or high-risk (HR-) HPV, e.g., HPV16 [4]. HR-HPV infections have been shown to cause anogenital and oropharyngeal cancers [5,6]. Infections of the skin with β-HPV (e.g., HPV8) generally occur in early childhood, when the virus becomes part of the normal microbiological skin flora. In immunocompetent individuals, persistent HPV infections of the skin are well controlled and are mostly asymptomatic. However, infections can become symptomatic in immunocompromised patients (e.g., immunosuppressed organ transplant recipients) and in patients with the rare genetic disorder epidermodysplasia verruciformis. These patients carry high β-HPV DNA loads on their skin and can develop β-HPV-mediated squamous cell carcinoma (SCC) [7,8,9].

After the initial infection of basal cells with HPV, a low rate of viral episome replication, coordinated by the viral proteins E1 and E2, allows the virus to evade recognition by the immune system [10,11,12]. The HPV proteins E6 and E7 are described as major oncoproteins because of their interference with key cell cycle regulators [13,14].

The immunorecognition of invading pathogens such as viruses usually occurs via so-called pattern recognition receptors (PRRs), which function as sensors for the innate immune system. These receptors detect unique molecular structures that are characteristic of pathogens, which are collectively referred to as pathogen-associated molecular patterns (PAMPs). The PRRs include Toll-like receptors (TLRs), RIG-I-like receptors (RLRs), and the cyclic GMP-AMP synthase (cGAS)-stimulator of interferon genes (STING) (cGAS/STING) [15]. TLRs are either found on the cell surface (e.g., TLR2, TLR4) or located in intracellular compartments, such as the endolysosome (e.g., TLR7, TLR8, TLR9) or the cytosol (e.g., retinoic acid inducible gene I (RIG-I) and melanoma differentiation-associated protein 5 (MDA5)) [16]. TLR3 is present on the cell surface of non-immune cells, such as epithelial cells and fibroblasts, as well as in the endolysosome of immune cells [17,18]. TLR3 senses long double-stranded (ds) RNA, TLR7/8 sense RNA degradation products, and TLR9 senses DNA containing unmethylated CpG motifs [15,19,20,21,22,23]. RIG-I is activated by double-stranded RNA with a blunt end and an uncapped 5′-triphosphate [24,25,26], and MDA5 recognizes longer dsRNA (>300 bp, e.g., high-molecular weight (HMW) poly(I:C)) [27,28,29,30,31]. However, the exact motif detected by MDA5 remains to be defined [23]. The cGAS/STING pathway is the main cytosolic type I interferon (IFN)-inducing dsDNA-sensing immune pathway [32,33,34,35]. The cGAS protein binds to dsDNA in a sequence-independent manner and induces the formation of the cyclic dinucleotide 2′3′cGAMP, which, in turn, triggers STING and leads to type I IFN secretion via TANK-binding kinase 1 (TBK1) and interferon regulatory factor 3 (IRF3) activation [34,36].

For HPV16 and HPV18, several interactions with PRR signaling pathways have been reported [37]. In keratinocytes, lower expressions of *RIG-I*, *MDA5*, and *TLR3* have been observed in the presence of HPV16 and HPV18 [38]. HPV16-*E6* forms a complex with TRIM25, which inhibits the ubiquitination of RIG-I and its CARD-dependent interaction with the mitochondrial antiviral-signaling protein (MAVS), thereby suppressing the RIG-I-mediated induction of type I IFN, chemokines, and IFN-stimulated genes (ISGs) [39]. Furthermore, HPV16-*E6* inhibits IRF3 transcriptional activity [40]. HPV16-*E7* impairs the activation of IRF1 [41,42,43], suppresses TLR9 transcription [44], and hijacks NLRX1 to induce the degradation of STING [45]. Additionally, HPV18-*E7* functions as an antagonist in DNA sensing by inhibiting the cGAS/STING pathway [46]. HPV16-*E7* and HPV18-*E7* induce the methyltransferase SUV39H1, leading to the epigenetic silencing of *cGAS*, *STING*, and *RIG-I* [47,48].

However, thus far, information about the interaction of β-HPV genes with innate immune receptors is limited. In the respective transgenic animals, our group recently confirmed the oncogenic potential of the HPV8 proteins E2, E6, and E7 [49,50,51]. In primary human keratinocytes, these viral proteins are able to downregulate IL-8 secretion [52]. In addition, HPV5-*E6* and HPV8-*E6* form complexes with TRIM25 [39], and HPV38-*E6* and -*E7* were found to inhibit the expression of *TLR9* [53].

In the present study, we investigated the expression and function of nucleic-acid-sensing PRRs upon stimulation with specific nucleic acid ligands in the presence or absence of HPV8 genes. We identified two HPV8 early proteins that negatively interfere with nucleic acid sensing.

## 2. Materials and Methods

### 2.1. Cell Culture

All cells were cultured at 37 °C and 5% CO_2_. Primary human skin keratinocytes (PHK) were obtained from Lonza Biosciences (Cat. No. 00192907, Lot No 0000188311, Lonza, Cologne, Germany) and grown in keratinocyte growth medium 2 (PromoCell, Heidelberg, Germany, #C-20011) with 1% penicillin/streptomycin (PS, Thermo Fisher Scientific, Waltham, MA, USA, #15140130). U2OS cells were passaged in IMDM (Thermo Fisher Scientific, Waltham, MA, USA, #12440053) containing 10% fetal calf serum (FCS) (Life Technologies, Carlsbad, CA, USA, #10270106) and 1% PS. The U2OS empty vector (pBabe neo) and U2OS-HPV8 cells were kindly provided by Mart Ustav (University of Tartu, Tartu, Estonia), and are described elsewhere [54]. The production of retroviruses and the retroviral transduction of human cells with recombinant retroviruses coding for HPV early proteins were carried out as previously described [55]. Briefly, the cells were seeded in 6 cm dishes. On the next day, retroviral supernatants were mixed in the presence of 5 μg/mL of hexadimethrine bromide (polybrene) and added to the cells. A spin infection was performed by centrifugation for 1 h at 300× *g*. The cells were then washed with PBS and cultured for another 2 days. Then, the cells were selected by adding G418 (500 μg/mL) until only the infected cells survived. In subsequent experiments, we used pooled stable cell populations to minimize possible variations due to the randomness of the viral integration site in the cellular chromosomes. The generation of pLXSN-based retroviral vectors coding for HPV8-*E1*, -*E2*, -*E6*, -*E7*, or -*E6/E7* and the confirmation of early gene expression by RT-qPCR have been previously described [56,57,58,59]. The construction of pLXSN vectors for HPV16-*E6*, -*E7*, and -*E6/E7* is described elsewhere [56,57,58].

Peripheral blood samples from healthy donors were obtained after written informed consent and institutional review board approval (University of Bonn, grant no. 007/17 and 516/20, Bonn, Germany). Peripheral blood mononuclear cells (PBMCs) were isolated by density gradient centrifugation and cultured in RPMI (Thermo Fisher Scientific, Waltham, MA, USA #21875091) containing 10% FCS and 1% PS.

### 2.2. RNA Isolation and qPCR Analysis

A total of 300,000 cells/well were seeded in 6-well plates, and 24 h later, the cells were harvested. After the total RNA preparation of two pooled wells, using the RNeasy Mini Kit (Qiagen, Hilden, Germany, #74106), a DNAse digestion was performed (Thermo Fisher Scientific, Waltham, MA, USA #EN0521), and cDNA synthesis was executed with the SuperScript VILO cDNA Synthesis Kit (Invitrogen, Carlsbad, CA, USA, #11754050). The cDNA served as the template for a qPCR analysis (Biobudget, Krefeld, Germany, #80-5805000) using gene-specific primers—hRIG-I (forw: 5′-GAAAGACTTCTTCAGCAATGTCC; rev: 5′-GTTCCTGCAGCTTTTCTTCAA), hMDA5 (forw: 5′-GGAGTCAAAGCCCACCATCT; rev: 5′-TGCCACTGTGGTAGCGATAA), hTLR3 (forw: 5′-CAGCTGACTAGGAACTCCTTT; rev: 5′-GGCTATGTTGTTGTTGCTTAGA), hSTING (forw: 5′-TGGGCTGGCATGGTCATATT; rev: 5′-CCCCGTAGCAGGTTGTTGTA), hGAPDH (forw: 5′-CACCATCTTCCAGGAGCGAG; rev: 5′-GTGCAGGAGGCATTGCTGA)—and the QuantStudio 5 Real-Time PCR System (Thermo Fisher Scientific, Waltham, MA, USA). Mean dCT levels were calculated by subtracting the CT value of GADPH from the CT value of the gene of interest. Relative expression levels were calculated by the 2^(−ddCT) method.

### 2.3. Measurement of Cytokine/Chemokine Secretion

A total of 20,000 cells/well were seeded in 96-well plates. Twenty-four hours later, the cells were transiently transfected in duplicate, with 500 ng/mL of control RNA (CA21 5′-CACACACACACACACACACAC; Biomers, Ulm, Germany), 3pRNA (IVT4 as described in [60]), HMW poly(I:C) (InvivoGen, San Diego, CA, USA, #tlrl-poly(I:C)), or Y-DNA (5′-GGGTATATATATGCATATATATAGGG, kindly provided by Prof. Martin Schlee, University of Bonn, Germany) using Lipofectamine 2000 (Thermo Fisher Scientific, Waltham, MA, USA, #11668019) or were treated by adding 10 µg/mL of HMW poly(I:C) to the cell culture medium. These ligand concentrations have been used by our group in a previous study [61] and also resulted in sufficient cytokine secretion and cell viability in the human keratinocytes used in the current study. A total of 400,000 PBMCs/well were seeded in 96-well plates and were subsequently transfected in duplicate with 500 ng/mL of DNAse-digested total RNA derived from HPV8 or HPV16 positive PHKs using Lipofectamine 2000. Twenty-four hours post-transfection, the cell-free cell culture supernatants were collected and analyzed using the human IP-10/CXCL10 (BD, Franklin Lakes, NJ, USA, #550926), IL-6 (BD, Franklin Lakes, NJ, USA, #555220), or IFN-a (Thermo Fischer Scientific, Waltham, MA, USA, #BMS216C-1MG, #BMS216MSTK, #BMS216MSTS) ELISA Set, according to the manufacturer’s instructions. For type I IFN analysis, 50,000 HEK-Blue^TM^ IFNa/b reporter cells/well (InvivoGen, Toulouse, France, #hkb-ifnab) were incubated with 20 µL of the cell culture supernatant or with 20 µL of a serial dilution of hIFNa2a (Miltenyi Biotech, Cologne, Germany, #130-094-116) as a standard. Twenty-four hours later, the supernatants were collected and mixed 1:1 with pNPP substrate, which was prediluted to a final concentration of 1 mg/mL (pNPP powder (Sigma Aldrich, St. Louis, MO, USA, #71768-5G) and dissolved to a concentration of 10 mg/mL in pNPP substrate buffer (100 mM Tris; 100 mM NaCl, 5 mM MgCl_2_; pH 9.5) in a 96-well plate. Approximately 15–30 min later (or when the lowest standard dilution reached a value of 1.0), the optical density was measured at 405 nm, followed by a calculation of the cytokine concentration based on a standard curve. ELISAs and type I IFN reporter assays were measured using the BioTek Epoch microplate spectrophotometer (BioTEK/Agilent, Santa Clara, CA, USA).

### 2.4. Cell Viability

To determine cell viability, MTT assays were performed by adding 10% (*v*/*v*) MTT/well (Carl Roth, Karlsruhe, Germany, #4022.2) to the 96-well plates used for cytokine/chemokine analysis after removal of the supernatant. Four to six hours after the addition of MTT, 10% SDS/well was added (final concentration 5%/well), and the cells were incubated overnight at 37 °C and 5% CO_2_, followed by an OD measurement using the BioTek Epoch microplate spectrophotometer (BioTEK/Agilent, Santa Clara, CA, USA. Since cell viability (measured by MTT assay (Figures S1 and S2)) was already reduced in the CA21-transfected cells, compared with the media control, and varied depending on the transfected ligand, all measured values were normalized to the percentage of living cells. For this purpose, all MTT values of a single experiment were divided by those of the respective empty vector or HPV8 CA21 MTT value, allowing for the generation of a “living cell normalization factor”. Subsequently, all cytokine/chemokine raw values from this experiment were divided by this “living cell normalization factor” to account for the different cell viabilities. The same calculations were performed for the untransfected poly(I:C) values, with the difference that the MTT value of the medium control was used instead of the CA21 value, since in this case stimulation was carried out without transfection.

### 2.5. Statistical Analysis

The graphs show the mean, as well as the standard error of the mean, unless otherwise stated. Statistical analysis was performed using a two-sided Student’s *t*-test followed by the Bonferroni method to correct for multiple testing. * indicates *p* < 0.05, ** *p* < 0.01, and *** *p* < 0.001.

## 3. Results

### 3.1. Expression Levels of RIG-I, MDA5, TLR3, and STING Are Downregulated by HPV16-*E6* but Not by HPV8 Early Proteins

**Figure 1:**
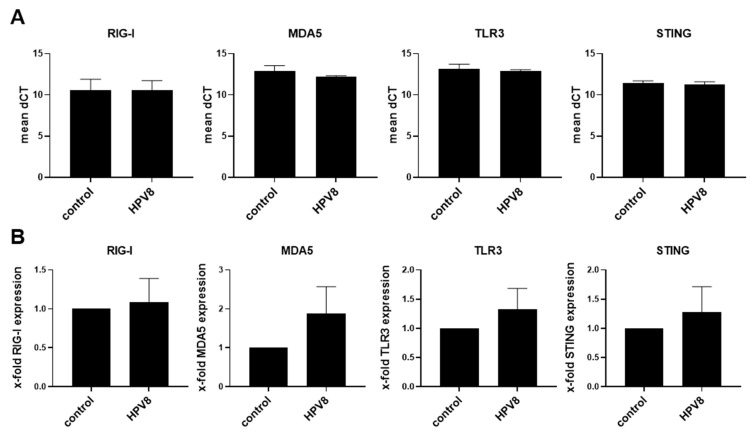
Expression levels of *RIG-I*, *MDA5*, *TLR3*, and *STING* in U2OS cells are not affected by the presence of HPV8. RNA from U2OS cells ± HPV8 was harvested, and *RIG-I*, *MDA5*, *TLR3*, and *STING* mRNA expressions were measured by RT-qPCR using gene-specific primers. Depicted are (**A**) the mean dCT values and (**B**) the x-fold expression levels ± SEM (*n* = 3).

**Figure 2:**
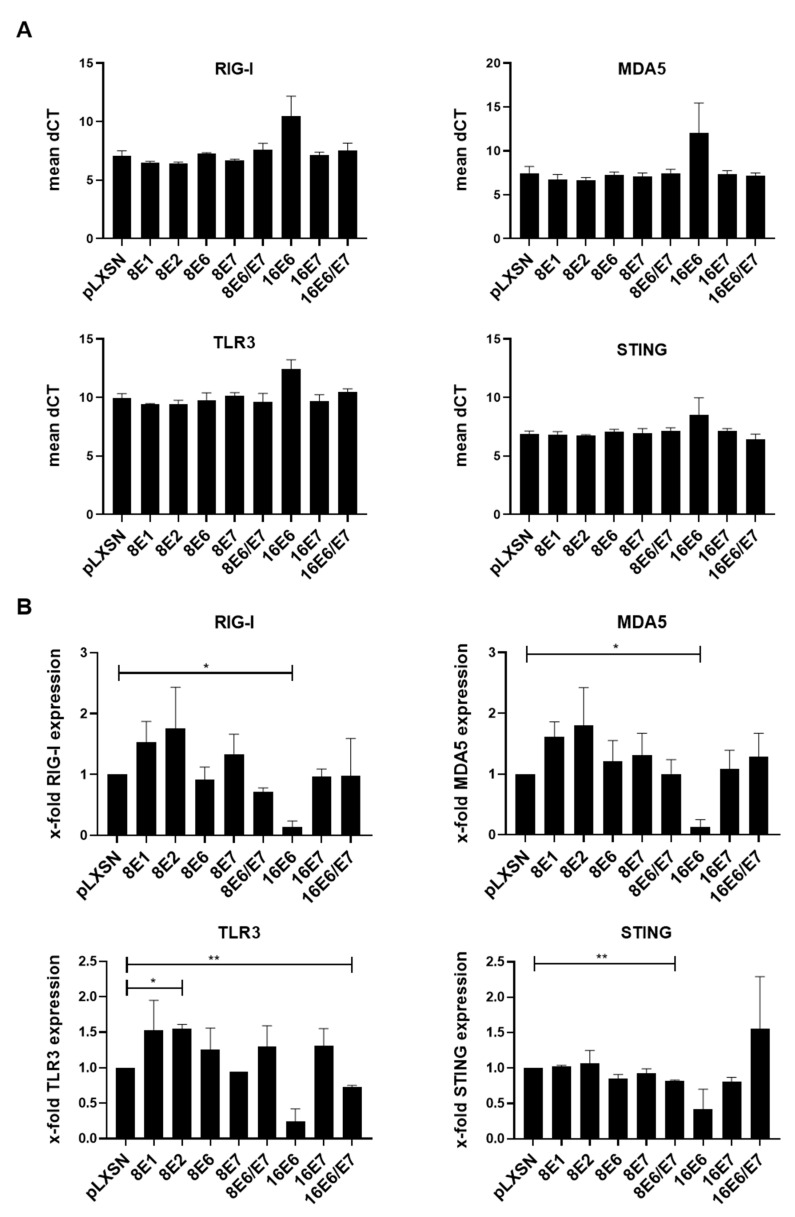
Expression levels of *RIG-I*, *MDA5*, *TLR3*, and *STING* in primary keratinocytes expressing HPV8 or HPV16 early proteins. RNA from primary skin keratinocytes stably transduced with empty vector (pLXSN) or vectors coding for distinct HPV8 or HPV16 early genes was harvested, and *RIG-I*, *MDA5*, *TLR3*, and *STING* mRNA expressions were measured by RT-qPCR. Depicted are (**A**) the mean dCT values and (**B**) the x-fold expression levels ± SEM (*n* = 2). * *p* < 0.05, and ** *p* < 0.01.

We used U2OS-HPV8 cells and primary human keratinocytes (PHK) to dissect the effect of the HPV8 early genes on nucleic acid sensing. Previously, the U2OS cell line, which is derived from a moderately differentiated osteosarcoma, was shown to efficiently support HPV8 DNA replication and gene expression in comparison to C33A, HEK293, or HaCaT cells [54,62]. First, we analyzed the expression of nucleic-acid-recognizing PRRs in both cell types by RT-qPCR using gene-specific primers. Empty vector (control) U2OS cells showed expression of *RIG-I*, *MDA5*, *TLR3*, and *STING*. These expression levels were not significantly altered in the presence of HPV8 (Figure 1A,B). The PHKs were also found to express *RIG-I*, *MDA5*, *TLR3*, and *STING*. The presence of HPV16-*E6* but not of HPV16-*E7* in PHKs resulted in a significant downregulation of the receptors *RIG-I* and *MDA5*, with a similar but non-significant trend for *TLR3* (*p* = 0.052) and *STING* (*p* = 0.174) (Figure 2A,B). PHK cells expressing HPV8-*E2* showed a higher baseline expression of *TLR3* (with a similar trend for *RIG-I* and *MDA5*). The presence of HPV8-*E1*, HPV8-*E6*, or HPV8-*E7* had no significant effect on the mRNA levels of *RIG-I*, *MDA5*, *TLR3*, and *STING* in PHKs. The expression of HPV8-*E6E7* led to a slight but significant reduction in *STING* mRNA levels (Figure 2A,B). *TLR7*, *TLR8*, and *TLR9* were not detectable in both cell types (data not shown).

### 3.2. MDA5-Induced Cytokine Secretion in U2OS Cells Is Diminished in the Presence of HPV8

**Figure 3:**
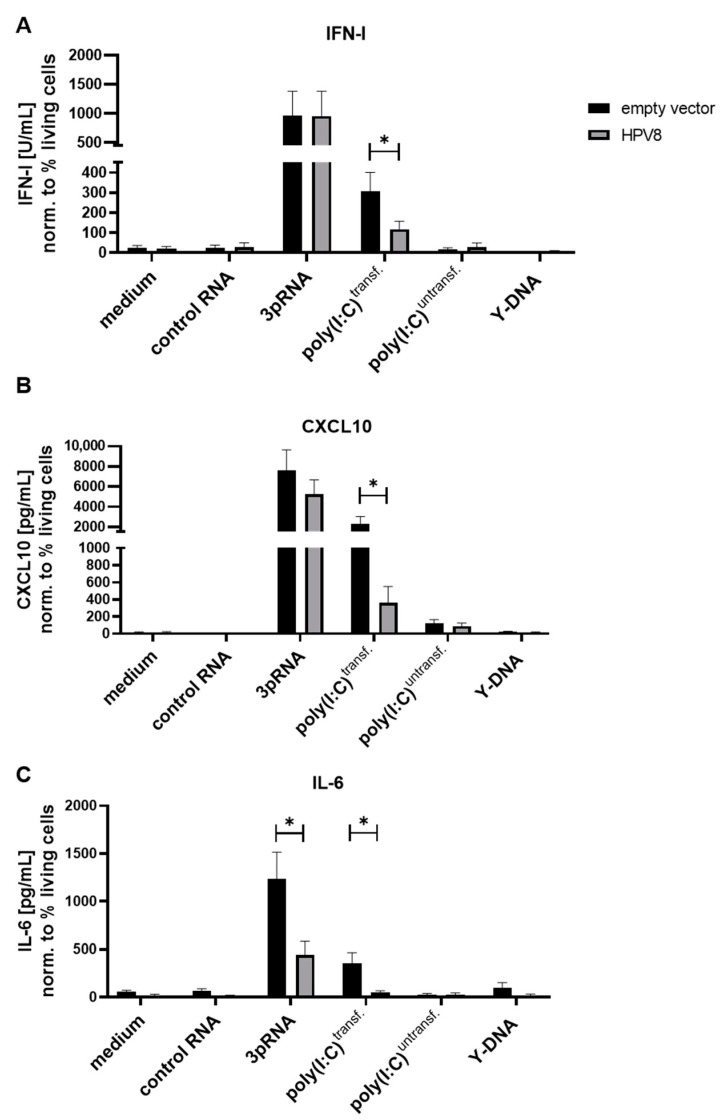
Poly(I:C)-induced cytokine secretion in U2OS cells is diminished in the presence of HPV8. U2OS cells ± HPV8 were transfected with control RNA, 3pRNA (RIG-I agonist), Y-DNA (STING agonist), or poly(I:C) (MDA5 agonist) or treated with untransfected poly(I:C) (TLR3 agonist). Twenty-four hours post-treatment, the cell culture supernatants were collected and cytokine secretion was measured using either (**A**) type I IFN reporter cells or by means of (**B**) CXCL10 or (**C**) IL-6 ELISA. Cytokine/chemokine levels are presented as the mean ± SEM normalized to the percentage of living cells to account for variations in cell viability due to the different stimuli used (*n* ≥ 3). * *p* < 0.05.

We next analyzed whether HPV8 interferes with PRR signaling. To this end, U2OS cells ± HPV8 were transfected with 3pRNA (RIG-I ligand) and poly(I:C) (MDA5 ligand) and were stimulated with non-formulated poly(I:C) (TLR3 ligand) or transfected with control RNA CA21. The 3pRNA, transfected poly(I:C), and, to a much lower degree, untransfected poly(I:C) induced type I IFN, CXCL10, and IL-6. The transfection of Y-DNA had no effect, which is consistent with previous reports of defective cGAS/STING signaling in U2OS cells [63]. The presence of HPV8 diminished the type I IFN, CXCL10, and IL-6 secretion induced by the transfection of poly(I:C) (MDA5 ligand), as well as the IL-6 secretion induced by 3pRNA (RIG-I ligand) (Figure 3A–C).

### 3.3. Expression of HPV8-E1 and HPV8-E2 Suppress MDA5-Induced Cytokines in Primary Human Keratinocytes

**Figure 4:**
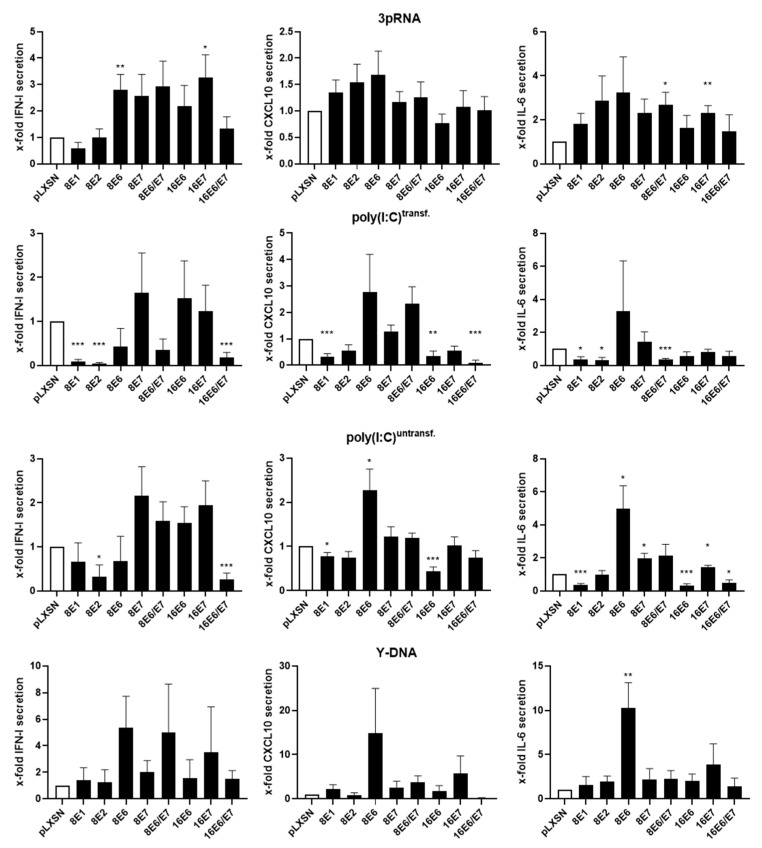
Expression of HPV8-*E1* and HPV8-*E2* decreases poly(I:C)-induced cytokine secretion in primary keratinocytes. Human primary keratinocytes, stably transduced with retroviral vectors expressing either the empty vector (pLXSN), HPV8 early genes, or HPV16 early genes, were transfected with 3pRNA (RIG-I ligand), Y-DNA (STING ligand), or poly(I:C) (MDA-5 ligand) or were stimulated with untransfected poly(I:C) (TLR3 ligand). Twenty-four hours post-stimulation, the cell culture supernatants were harvested. Type I IFN expression was analyzed using reporter cells. CXCL10- and IL-6 secretion was measured by ELISA. Cytokine/chemokine levels are depicted as x-fold secretion ± SEM, normalized to the percentage of living cells to account for variations in cell viability due to the different stimuli used (*n* ≥ 3). * *p* < 0.05, ** *p* < 0.01, and *** *p* < 0.001.

To confirm HPV8-mediated PRR inhibition, and to identify specific viral early proteins that interfere with PRR signaling, we analyzed the induction of cytokines in PHKs that were stably expressing different genes of HPV8 or HPV16. The combined expression of HPV16-*E6* and HPV16-*E7* early genes potently suppressed type I IFN and CXCL10 induced by transfected poly(I:C) (MDA5 ligand), but not by 3pRNA (RIG-I ligand) (Figure 4), which is in accordance with published data suggesting that both HPV16-*E6* and HPV16-*E7* are required for the suppression of IFN-induced genes [64]. Moreover, individually expressed HPV8-*E1* and HPV8-*E2* were each shown to potently suppress type I IFN and IL-6 secretion when induced by transfected poly(I:C) (Figure 4). Together, these results indicate that type I IFN induction by MDA5, but not by RIG-I, is consistently suppressed, not only by combined HPV16-*E6*/E7 but also by HPV8-*E1* or HPV8-*E2* alone.

### 3.4. PBMCs Secret Elevated Cytokine Levels upon Stimulation with Total RNA Isolated from HPV8-E2-Expressing Keratinocytes

**Figure 5:**
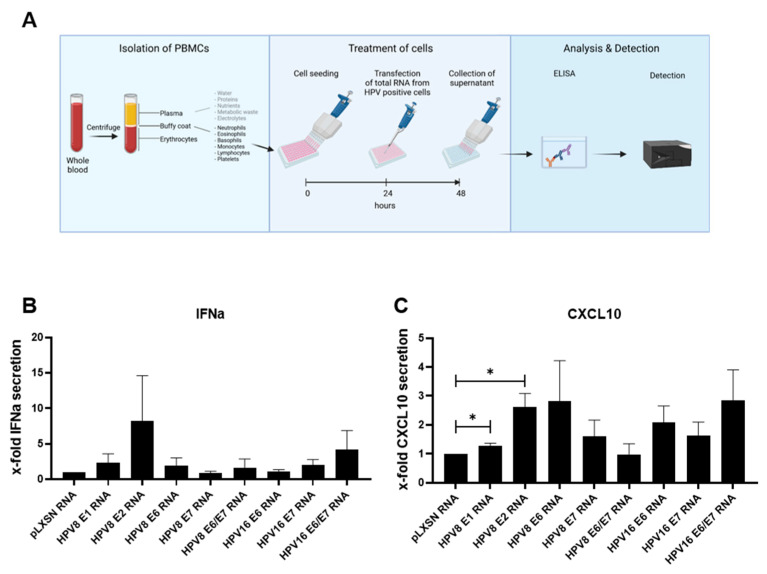
PBMCs secrete elevated cytokine levels upon stimulation with total RNA isolated from HPV8-*E2*-expressing keratinocytes. Total RNA from primary keratinocytes expressing distinct HPV8 or HPV16 genes was collected and transfected into PBMCs from six individual donors. Supernatants were harvested 24 h post-transfection, and IFN-a (**B**) and CXCL10 (**C**) were analyzed by ELISA, as shown in the schematic assay workflow (created with BioRender.com (accessed on 3 May 2022, (**A**)). Presented is the x-fold IFN-a or CXCL10 secretion ± SEM normalized to the values obtained with RNA isolated from pLXSN-expressing cells. * *p* < 0.05.

Based on the observation that U2OS-HPV8 cells and HPV8-*E1* and HPV8-*E2*-expressing PHKs exhibit diminished PRR signaling, we wondered whether HPV8-positive cells might contain viral RNA that is detected by PRRs. To address this, human peripheral blood mononuclear cells (PBMCs) were transfected with DNase-treated total RNA that was isolated from PHKs expressing specific HPV8 or HPV16 genes, and cytokine secretion was measured 24 h post-transfection. We found that the transfection of total RNA isolated from HPV8-*E2* (and, to some degree, HPV8-*E1*)-expressing PHK cells increased type I IFN and CXCL10 levels in PBMCs (Figure 5A,B), indicating that HPV8-*E2* PHK cells contain an RNA ligand that is detected by nucleic-acid-sensing PRRs.

## 4. Discussion

Due to the coevolution of pathogens with their hosts, viruses have evolved evasion strategies to escape or inhibit the host’s immune system in order to replicate and propagate in infected cells. In this study, we aimed to identify viral genes that might be involved in the escape of the β-HPV type HPV8 from the detection of viral nucleic acids by the innate immune system. We demonstrated that U2OS cells and PHKs express the nucleic-acid-sensing PRRs *RIG-I*, *MDA5*, *TLR3*, and *STING*, but not *TLR7*, *TLR8*, and *TLR9*. We found that in PHKs, HPV16-*E6* downregulated the expression of *RIG-I*, *MDA5*, *TLR3*, and *STING* and, in conjunction with HPV16-*E7*, effectively suppressed the type I IFN response to MDA5 activation. Furthermore, in this experimental setting, we identified HPV8-*E1* and HPV8-*E2*, which are also able to antagonize MDA5 activation.

Consistent with our findings, previous studies have demonstrated the expression of *RIG-I*, *MDA5*, *TLR3*, and *cGAS/STING* in the human epidermis, in PHKs, and in the skin-derived HaCaT cell line, as well as in U2OS cells [18,63,65,66]. Furthermore, the absence of *TLR7* and *TLR8* expression in U2OS cells and PHKs in our study is in agreement with the literature [67,68]. We did not detect *TLR9* in either of these cell types. The literature on *TLR9* expression remains controversial [53,65,67,68]. One possible explanation for inconsistent results regarding *TLR9* expression in primary keratinocytes might be the differing cell culture conditions used in those studies.

HPV16-*E6* mediated a reduction in *RIG-I*, *MDA5*, *TLR3*, and *STING* expression in PHKs in our study, which is in line with the study by Reiser et al. (2011), who described considerably lower *RIG-I*, *MDA5*, and *TLR3* expression levels in HPV16- and HPV18-positive keratinocyte cell lines but did not report the responsible viral proteins [38]. Our data provide evidence that the HPV16-*E6* protein is responsible for the downregulation of these PRRs. Unlike HPV16-*E6*, HPV8 proteins did not cause a reduced expression of *RIG-I*, *MDA5*, *TLR3* and *STING*. This indicates that the functional interference of HPV8 with these receptors is not on the transcriptional level.

The stimulation of U2OS cells and PHKs with nucleic acid ligands induced type I IFN, CXCL10, and IL-6, demonstrating that the corresponding receptors activate anti-viral and pro-inflammatory signaling pathways. The presence of HPV8-*E1* and HPV8-*E2*, but not HPV8-*E6* or HPV8-*E7*, substantially diminished poly(I:C)-induced type I IFN, CXCL10, and IL-6 secretion. The downregulation of the cytokines induced by transfected poly(I:C) (targeting MDA5) was robust, while the effects on activation by untransfected poly(I:C) (targeting TLR3) were more variable. No consistent effect of HPV8-*E1* and HPV8-*E2* was observed on cytokine induction by RIG-I or by STING. HPV8-*E1* and HPV8-*E2* are expressed very early during the viral life cycle and are important for viral genome amplification. Accordingly, it is beneficial for the virus if these replication factors also mediate immune evasion. Interestingly, E1 of HPV11, HPV16, and HPV18 have been shown to downregulate immune-response genes, including *IFNb1*, *IFN-lambda1*, *CCL5*, and *RSAD2/Viperin* [69]. In cells expressing HPV11-*E1*, HPV16-*E1*, or HPV18-*E1*, the expression of *IFNb1* and *IFN-lambda1* was also shown to be diminished after stimulation with poly(I:C) [69]. The transduction of recombinant adenoviruses that contain HPV16-*E2* or HPV18-*E2* downregulated *STING* and *IFN-k*. In that study, the authors also provide evidence that the amino-terminal domain (TAD) of E2 plays an essential role in the downregulation of *STING* and *IFN-k* [70]. Recently, HPV16-*E2* has been observed to downregulate U-ISGF3 genes in N/Tert cells, such as *DDX58* (*RIG-I*), *IFIT1* (*ISG56*), *IFIT3* (*ISG60*), *IRF7*, *MX1/2*, and *OAS1/2/3*, amongst others [71,72]. With our study, we can add HPV8-*E1* and HPV8-*E2* to the list of immune-escape-mediating viral proteins.

Unlike for the MDA5 pathway, we observed no major inhibition of RIG-I-dependent signaling by HPV8, except for some inhibition of the proinflammatory cytokine IL-6. Therefore, one could speculate that there is no need for HPV8 to suppress RIG-I recognition. On the other hand, Chiang et al. (2018) described an interaction of HPV8-*E6* with TRIM25 that led to the suppression of the RIG-I-mediated induction of IFN-b, chemokines, and IFN-stimulated genes (ISGs) [39]. Furthermore, as RIG-I and MDA5 pathways share downstream signaling molecules, a potential HPV8-dependent inhibition of MDA5 is likely to occur through the post-translation modification of MDA5 itself, and/or of MDA5-regulating proteins [73,74]. The exact mechanism of suppression warrants further investigation.

Our finding that total cellular RNA derived from HPV8-*E2*-expressing keratinocytes induces antiviral cytokines indicates that either the HPV8-*E2* RNA itself is immune stimulatory or that HPV8-*E2* triggers the immunorecognition of endogenous RNA. The HPV8-*E2* ORF contains a putative promoter for RNA polymerase III [75], which might trigger the formation of Pol III-derived small RNAs. Two HPV8 sequences that are 53 bp apart correspond almost perfectly to boxes A and B of the consensus tRNA gene promoters. T-rich sequences located 71 and 218 bp downstream of the B box have been described, and these may serve as transcriptional terminators [75]. Therefore, one could speculate that HPV8-*E2* ORF-derived RNAs indeed activate cytoplasmic nucleic-acid-sensing receptors.

In conclusion, the newly identified HPV8-*E1*- and HPV8-*E2*-mediated immune evasion may represent an important component of viral activities that enable β-HPV to establish a persistent viral infection in the skin. A detailed knowledge of the β-HPV-associated immune evasion mechanisms will form a solid basis for the development of innovative therapeutic strategies and vaccine approaches to tackle β-HPV-associated skin cancer.

## Data Availability

Data is contained within the article or Supplementary Materials.

## Supplementary Materials

The following supporting information can be downloaded at: https://www.mdpi.com/article/10.3390/v14071361/s1, Figure S1: Cell viability of U2OS cells after PRR ligand treatment; Figure S2: Cell viability of primary human keratinocytes upon PRR stimulation with specific ligands.
