# Supplementary figures and images for: Human Beta Papillomavirus Type 8 E1 and E2 Proteins Suppress the Activation of the RIG-I-Like Receptor MDA5
Source: Viruses. 2022 Jun 22;14(7):1361. doi: 10.3390/v14071361 (PMC9317666; doi:10.3390/v14071361)

cell viability [% of control]

U2OS

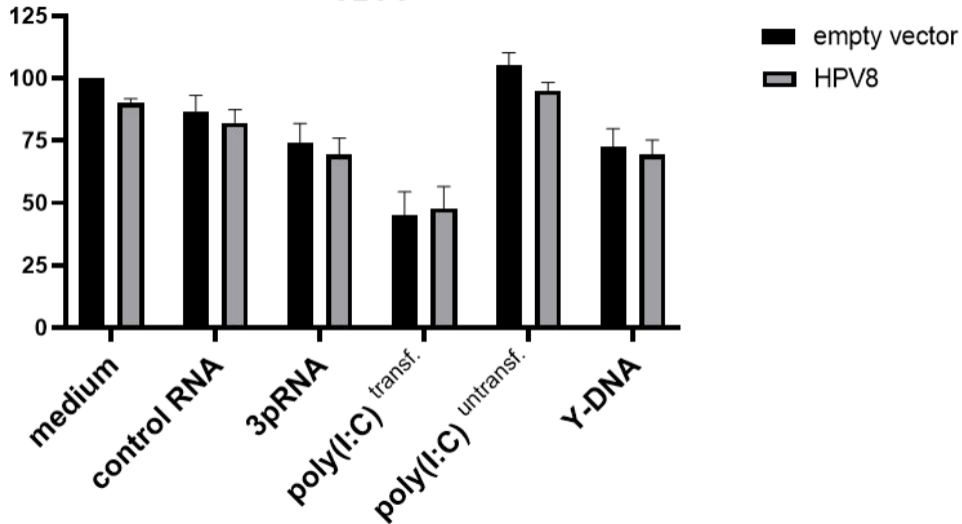

Supplement: Supplementary file 1 [file viruses-14-01361-s001.zip › Supplementary Figure S1.pdf]

medium

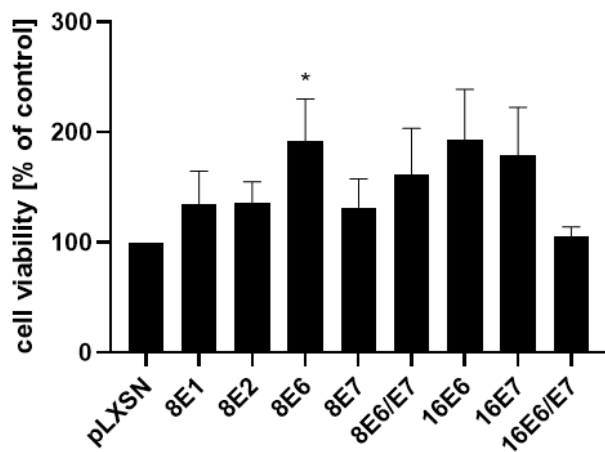

control RNA

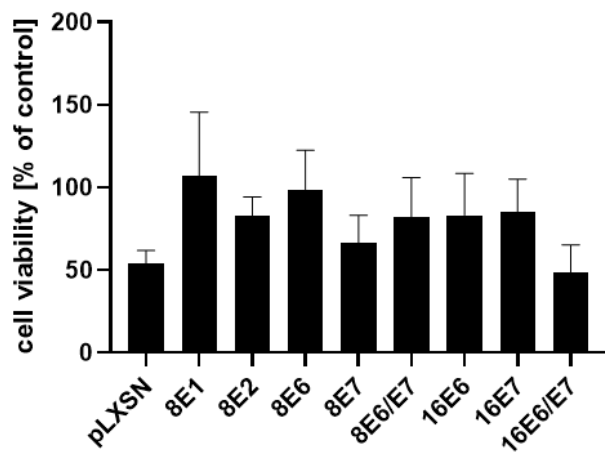

3pRNA

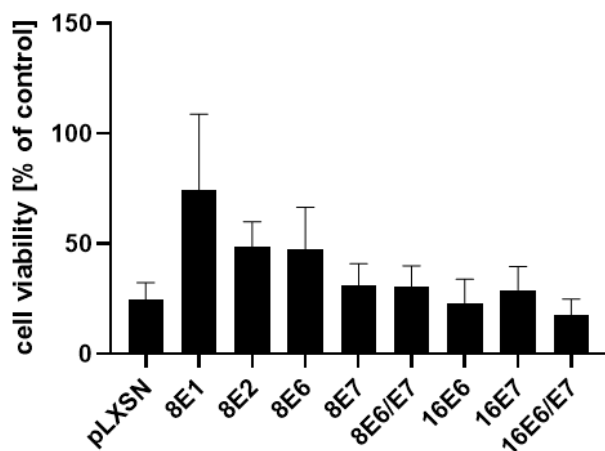poly(I:C)<sup>transf.</sup>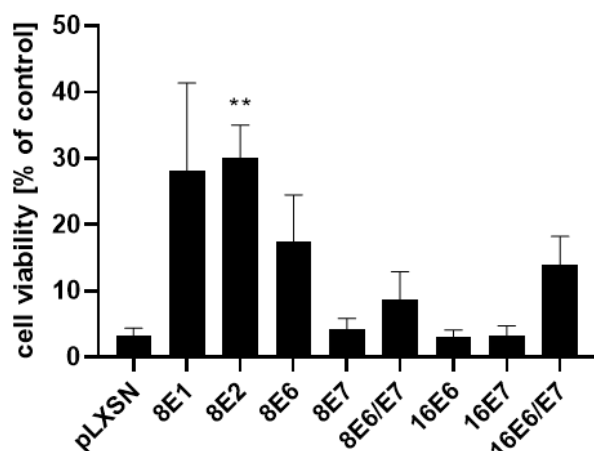poly(I:C)<sup>untransf.</sup>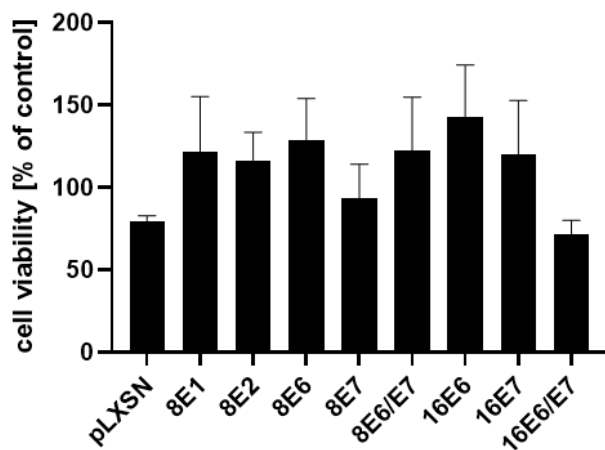

Y-DNA

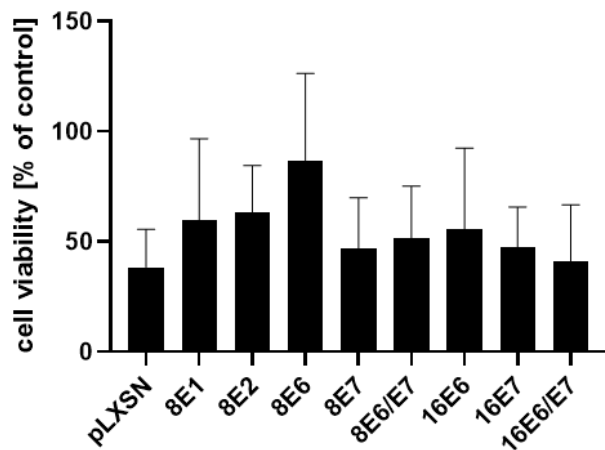

Supplement: Supplementary file 1 [file viruses-14-01361-s001.zip › Supplementary Figure S2.pdf]
